# Perceptions of dietary intake amongst Black, Asian and other minority ethnic groups in high-income countries: a systematic review of qualitative literature

**DOI:** 10.1186/s40795-023-00743-8

**Published:** 2023-07-13

**Authors:** Abimbola S. Ojo, Lawrence A. Nnyanzi, Emma L. Giles, Louisa J. Ells, Olusegun Awolaran, Sylvester R. Okeke, Agani Afaya, Obasanjo Afolabi Bolarinwa

**Affiliations:** 1 Centre for Public Health, School of Health & Life Sciences, University of Teesside, Teeside, UK; 2 Obesity Institute, School of Health, Leeds Beckett University, Leeds, UK; 3 Department of Community Medicine, College of Medicine, University of Ibadan, Ibadan, Oyo State Nigeria; 4 Centre for Social Research in Health, UNSW Sydney, Sydney, Australia; 5 Mo-Im Kin Nursing Research Institute, College of Nursing, Yonsei University, 50‑1, Yonsei‑ro, Seodaemun‑gu, Seoul, Seoul, 03722 South Korea; 6 Department of Nursing, School of Nursing and Midwifery, University of Health and Allied Sciences, Ho, Ghana; 7 Department of Public Health, York St John University, London, UK; 8 Discipline of Public Health Medicine, School of Nursing and Public Health, University of KwaZulu-Natal, Durban, South Africa

**Keywords:** Perception, Dietary intake, Black, Asian and other minoritised ethnicities, High-income countries, Systematic review

## Abstract

**Background:**

Minority ethnic groups are a fast-growing population in many high-income countries, partly due to the increasing population of immigrants and second-generation migrants. The dietary practices of some of these minority ethnic groups might make them to be disproportionately affected by obesity and increase their risks of developing non-communicable diseases. Population-specific interventions and strategies are vital to addressing poor nutritional practices among this population. Thus, this study systematically reviewed the perceptions of dietary intake amongst Black, Asian and other minority ethnic groups in high-income countries.

**Methods:**

This systematic review was conducted in line with the guidelines of the Joanna Briggs Institute (JBI) methodology for systematic reviews, using a meta-aggregative design. This systematic review identified and synthesised qualitative literature on the perceptions of dietary intake amongst BlackAsian and other minority  ethnic groups in high-income countries. An extensive and comprehensive database search was conducted between January 2000 – May 2022 and included twenty (20) studies that met the eligibility criteria from six countries. The included studies were assessed for quality using the JBI qualitative assessment and review instrument. The JBI data extraction tools were used to retrieve relevant data from included articles, and the data were thematically analysed.

**Results:**

We identified eight major themes across this database: (1) “Social and Cultural Factors,” (2) “Availability and Accessibility,” (3) “Family and Community Influences,” (4) “Food Preferences”, (5) “Home Country Food Versus Host Country Food” (6) “Dietary Acculturation” (7) “Health and Healthy Eating” (8) “Perception of Nutritional Information.”

**Conclusion:**

Overall, Black, Asian, and other minority ethnic groups individuals were found to be aware of the effects of unhealthy eating on their health, and some of them have nutritional knowledge, but social and cultural factors, including structural factors, were deterrents to their healthy eating behaviours. An important finding from this review is that some participants believed that nutritional information, based on bio-medical science, was intended for only White population groups and that it was antagonistic to their cultural and community well-being.

## Background

The increasing prevalence of obesity and its related health consequences remain a public health concern in developed and developing countries [1]. According to the World Health Organization (WHO), nearly one-third of the global population is living with obesity or weight-related health concern [1, 2]. Globally, obesity prevalence has evolved to an epidemic level in the past three decades. Prior to now, obesity was associated with affluence and was prominent in high-income countries (HICs), but in recent times, it has crept into low-and middle–income countries (LMICs), resulting in a double burden of diseases as these countries try to address both communicable and non-communicable diseases (NCD) [3].

The past few decades have recorded an unprecedented influx of immigrants into HICs [4, 5], which according to the World Bank [6], are countries with a gross national income of US$12,056 or more per capita. This influx has contributed to a fast-growing population of first, and second-generation immigrants in their host countries [5–7]. In addition to the rising number of immigrants and second-generation immigrants in HICs, some of these countries have indigenous minority groups; for instance, the United States Census Bureau [6] classified United States (US) indigenous minority groups as Black or African, Hispanic, Latino, Asian or Indian Americans.

In comparison with the ethnic majority groups in HICs, Black, Asian and other minority ethnic communities are disproportionately affected by a higher risk of diet-related non-communicable diseases such as obesity, type 2 diabetes, and cardiovascular diseases [8–13]. For instance, in the United Kingdom, people from Black, Asian and other  minority ethnities have higher rates of Type 2 diabetes compared to the White British population [14]. Similarly, this pattern of adverse health outcomes was reported among Hispanic minority population living in the United States compared to the White population [15]. Futhermore, South Asian populations living in HICs are at higher risk of cardiovascular diseases due to diets high in saturated fats, sugar, and salt [16]. Studies also suggest that Black, Asian and other minority ethnic populations may have higher rates of obesity and hypertension compared to the White population in HICs [17–19].

This higher risk may be due to a complex and multifactorial relationship between ethnicity and obesity [8–10]. To reduce the prevalence of these diet-related non-communicable diseases, the WHO recommends promoting healthy lifestyles such as good physical activity and healthy eating behaviours. However, evidence indicates that healthy lifestyle resources are not well accepted and are underutilised among Black, Asian and other minority ethnic groups [20, 21].

There also exists a dearth of literature investigating the dietary pattern of Black, Asian and other minority ethnic populations. Most existing research has focused on the white ethnic group, with few studies on dietary acculturation and food components. Hence the difficulty in understanding the perception of this group with regard to their dietary intake and preferences [4, 20, 22, 23]. It is, therefore, not a surprise that policies and practices are informed by limited and contradictory evidence that does not tackle the issues of a healthy diet and obesity among these groups [20–22]. Therefore, there is a need for an in-depth understanding of the complexity of the Black, Asian and other minority ethnic groups’ dietary intake. Thus, this study systematically reviewed qualitative studies on dietary intake perceptions among Black, Asian and other minority ethnic communities in high-income countries. This study’s results may help inform recommendations and policies to promote healthy eating and weight among these population groups.

## Methods

This systematic review was conducted in line with the guidelines of the Joanna Briggs Institute (JBI) methodology for systematic reviews, using a meta-aggregative design [24]. This systematic review (PROSPERO - CRD42018116426) aimed to identify and synthesise qualitative literature on the perceptions of dietary intake amongst Black, Asian and other minority ethnic groups in high-income countries.

### Search strategy and data sources

An extensive search was conducted for data gathering using MEDLINE, CINAHL, British Nursing Index (BNI), ProQuest, PsycINFO, Cochrane Public Health Group Register, Web of Science, Cochrane Library, EMBASE, Nursing and Allied Health Sources, Applied Social Sciences Index and Abstracts (ASSIA), Social Care Online and SCOPUS, to include studies conducted between January 2000 and May 2022 as indicated in Table 1.

**Table 1:** Search strategy

**Keywords**
Perception | Dietary intake | Black, Asian, and Minority | High-income countries
**The search words**
Search: (((((((((((((black and ethnic minority) OR (bme) OR (minority groups) OR (ethnic minority) OR (blacks) OR (black afric**) OR (asian) OR (pakistani) OR (bangladeshi) OR (carribbean) OR (migrant*) OR (immigrant*) AND (dietary intake) OR (diet) OR (dietary pattern) OR (food) OR (dietary habits) OR (food habits) OR (dietary practice) OR (food practice) OR (eating behaviour) OR (healthy eating) AND (percept*) OR (attitude) OR (views) OR (opinion) OR (beliefs) OR (idea) OR (experience) OR (habit) OR (behaviour)
**Filter date**
from 2000/1/1 to 2022/5/30

The systematic review formatting structure, PICOTS (Population, Issue, Context, Outcome, Timing, and Study Type), was used to develop the review eligibility criteria, as shown in Table 2. Studies with the following criteria were included; (1) reported adults (18 years and over) from the Black, Asian and other minority ethnic population, (2) reported dietary intake, food perception, barriers to healthy eating resources, other unhealthy habits such as taking sugary drinks, irregular meal patterns and cooking patterns (3) was conducted in HICs (4) reported perception, belief, views around the dietary intake, healthy eating, use of healthy diet resources (5) was conducted using primary qualitative research including focus groups, interviews, ethnography, phenomenology, grounded theory, action research, feminist research e.t.c.  (6) were published in English language and between January 2000 and May 2022 (Table 1).

**Fig. 1:**
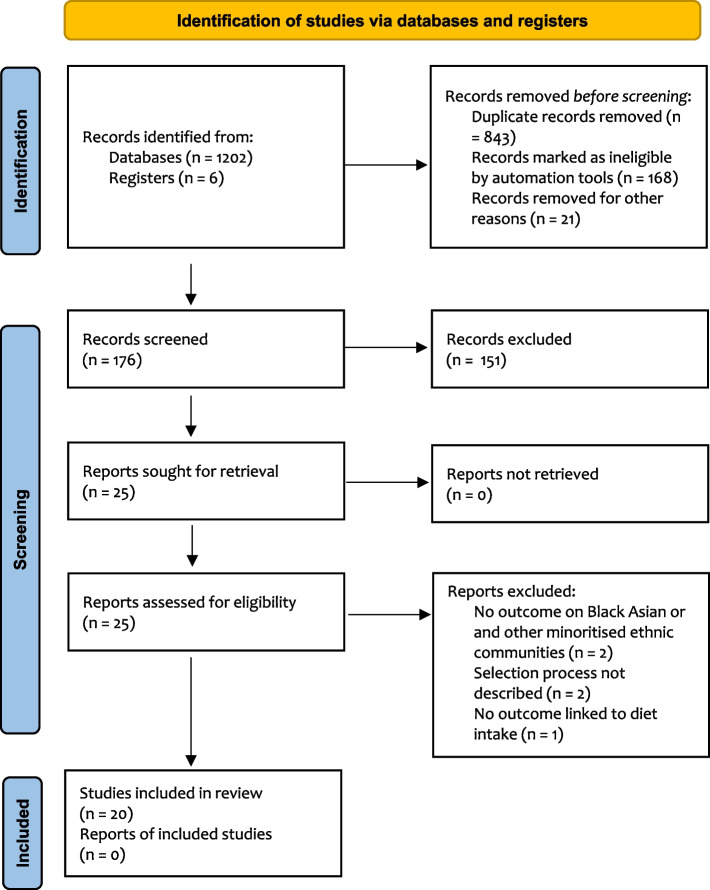
Search results using PRISMA flow chart [25]

The records retrieved from the databases were first screened, and duplicate records were removed. Afterwards, the titles and abstracts of the identified studies were screened for relevance and inclusion. Following the title and abstract screening, full-text articles were screened for inclusion. A hand search through the reference list of included articles was conducted, but no additional article was identified. The full article selection process is detailed in the PRISMA 2020 flow diagram (Fig. 1) [25]. The JBI [26] standardised data extraction tools (JBI-QARI) were used to retrieve relevant data from included articles. Data extraction was conducted independently by three reviewers (ASO, OAB, and OA), with 25% of the included articles checked by the fourth reviewer (EG ). Data extracted from the articles included specific details about the phenomena of interest (dietary intakes, food perception, barriers to healthy eating resources, and other related behaviours), populations, study methods, and outcomes of significance such as perception, belief, or views around the dietary intake, healthy eating, and use of healthy diet resources. Only data on adult participants were retrieved from papers that included adults, children, and adolescents.

**Table 2:** The components of the PICOS template

Population	Black, Asian and other minority ethnic Adults (18 years and older)
Issue	Dietary intake, food perception, food barriers, dietary knowledge, healthy diet resources
Context	High-income countries, according to the World Bank classification (The World Bank, 2018).
Outcome	Perception, beliefs, and view around the dietary intake, healthy eating, and use of healthy diet resources
Study type	Qualitative studies

### Data synthesis

In line with JBI’s [24] recommendations, data synthesis was done using the process of thematic analysis. The findings were classified as unequivocal, plausible, or unsupported according to their level of plausibility. Findings were deemed unequivocal if they were illustrated beyond a reasonable doubt. Findings deemed unrelated to corresponding illustrations were categorised as plausible, while findings not supported by data were deemed not credible. Only unequivocal findings illustrated by participants’ quotes were included in this meta-synthesis, even though a finding could be derived from either themes or metaphors from the data analysis, authors’ observations, and/or other analytical data [26]. However, findings in this meta-synthesis were limited to themes and metaphors that were extracted from corresponding quotations from the included articles. Findings were not reinterpreted; they were reported according to the original meanings presented in the included articles.

The findings extracted were read several times to make meaning, after which two independent reviewers undertook free line-by-line coding. The codes were constantly checked, compared, and discussed to resolve differences. The final codes were then reviewed and grouped into categories. The categories developed were further grouped according to their similarity of meaning, using the qualitative assessment and review instrument (JBI-QARI). Credibility was assigned to the pooled findings by the reviewer, and 25% of the articles were checked by the second reviewer (EG) for confirmation. A majority (16) of the categories were named verbatim according to the themes derived from the included articles; only four categories were renamed according to the meaning of the findings and the corresponding quotations (psychological factors, nutrition knowledge, lack of portion and traditional foods suitable for home country than host country).

### Assessment of methodological quality

Following the selection of articles that met the inclusion criteria, a quality assessment of 20 papers was conducted. To reduce the risk of bias and ensure the inclusion of high-quality studies in this review, selected articles were assessed independently by two reviewers (ASO and OAB) using the JBI (2015) standardised Qualitative Assessment and Review Instrument (JBI-QARI) [26]. The following criteria were applied for the methodological assessment of the included articles:

1. The congruity between the philosophical position adopted in the study, study methodology, study methods, representation of the data and the interpretation of the results.
2. The degree to which the biases of the researcher are made explicit.
3. The relationship between what the participants reported to have said and the findings of the research.

There were ten criteria used for the methodological appraisal of each article, providing a total of 10 scores, with ‘Yes’ allocated ‘1’ while ‘No’ and unclear allocated ‘0’. Any article that scored seven and (or) above was classified as high quality and was included. Although the JBI-QARI does not specify a cut-off point for the quality classification of the articles, the reviewer decided to select only high-quality articles, given that the aim is to use the findings of this review to develop recommendations to promote healthy eating and prevent obesity among Black, Asian and other minority ethnic communities.

## Results

### Search results and study selection

Following a systematic search, a total of 1202 studies were identified from the databases; however, based on the sifting of the titles and abstracts, assessment of the article’s full texts and methodological screening in line with the study inclusion and exclusion criteria, only 20 articles were finally included in this review (Fig. 1).

Table 3 shows the studies included in the review; 20 studies included employed different qualitative designs. The majority (*n* = 9) of the included studies used a focus group method [27–34]. Six studies used in-depth-interviews [35–40], with the remaining five studies using more than one qualitative method, being a combination of semi-structured or in-depth interviews with observations [41–45].

**Table 3:** Study characteristics of synthesised articles

Author, Year of Publication (20)	Country	Qualitative Methods	Participants’ Characteristics	Recruitment	Dietary Behaviour Measured
Antin and Hunt (2012) [41]	United States	Semi-structured, free list and card sorting activity, photo-elicitation activity (Multi-method qualitative)	African American young women (low-income) age range between 18–25 years, (*N* = 20)	Recruited via posters in the local community and online	Food choices
Beagan and Chapman (2012) [42]	Canada	Semi-structured, Observations (Ethnography)	African Nova Scotian (Indigenous African), aged 13 to 71 years (*N* = 13 families comprise of 38 participants, 14 youths, 22 adults, and 2 elders).	Recruited via advertisements, word of mouth, and snowballing.	Food practice and healthy eating
Blanchet et al. (2018) [35]	Canada	In-depth Interview (Exploratory qualitative study)	Sub-Sahara African and Caribbean immigrants’ mothers, aged between 30 to 45 years (*N* = 12)	Volunteers from the previous study were invited to take part in the present study.	Dietary acculturation
Chapman et al. (2011) [43]	Canada	Semi-structured interviews and observation (Ethnography)	Punjabi families, 13 youths, 19 adults and 7 elders (*N* = 39)	Purposeful sampling recruited families	Food practices and healthy eating
Garnweidner et al. (2012) [36]	Norway	In-depth interviews (Phenomenology)	African and Asian women immigrants, low-middle SES aged between 25–60 years (*N* = 21)	Purposeful sampling	Food habits and dietary acculturation
Jakub et al. (2018) [27]	United States	Focus groups (Ethnography)	Second-generational African immigrants aged 18–23 years (*N* = 20)	Recruited via community contacts and snowball via word-of-mouth	Food practices and healthy eating
James (2004) [46]	United States	Focus groups (Qualitative study)	African American men and women of low-middle SES, aged 18–69 years (*N* = 40)	Recruited through community contacts and asked for volunteers	Food choices
Koenig et al. (2012) [28]	United States	Focus groups (Ethnography)	Asian Indians, men and women, high SES (*N* = 15) Age not reported	Recruited via medical health records and self-identified individuals	Food practice and health
Lawrence et al. (2007) [29]	United Kingdom	Focus groups (Qualitative study)	African (Somalia, Zimbabwe), South Asian (Pakistani/ Bangladeshi) girls and young women aged 12-35yrs (*N* = 33)	Recruited via local network and contacts with community leaders	Food choices
Leu and Banwell (2016) [37]	Australia	In-depth interviews (Qualitative study)	Southeast Asian undergraduate students, males and females, aged 18 years and over (*N* = 31)	Recruited via South Asian students’ organisation, campus events and snowballing	Food preferences and behaviours
Mahadevan and Blair (2009) [44]	United States	In-depth interviews and observation (Grounded Theory)	Indians, vegetarians, men and women were aged 20–70 years (*N* = 28).	Friends and acquaintances of the lead researcher, social contacts, and snowballing	Dietary acculturation
Mellin-Olsen and Wandel (2005) [30]	Norway	Focus groups (Qualitative Study)	Pakistani immigrants’ women (*N* = 25) (Age not reported).	Recruited via the Oslo Health Study 2000–2001, using a purposive sampling method	
Mensah et al. (2022) [40]	United Kingdom	In-depth interviews (Phenomenology)	Afro-Caribbean, Asian, Latino and Hispanic Students aged 18–50 years (*N* = 43).	Recruited via university societies, chain referral sampling	Dietary acculturation
Mukherjea et al. (2013) [31]	United States	Focus groups (Exploratory)	Asian Indian immigrants, men and women, aged 45–84 years (*N* = 38)	Recruited via putting flyers at the community centres, Indian grocery stores, and faith-based organisations	Dietary beliefs and practices
Nicolaou et al. (2009) [32]	Netherlands	Focus groups (Qualitative study)	Turkish and Moroccan immigrants’ men and women aged = 20–40 years (*N* = 83)	Recruited via community centres, mosques, and organisations using leaflets and word of mouth	Dietary acculturation
Ochieng (2011) [38]	United Kingdom	In-depth Interviews (Qualitative study)	African Caribbean (eight men and ten women) aged 39–60 years (*N* = 18)	Recruited via Black churches and voluntary organisations using the purposive sampling method.	Dietary behaviours
Osei-Kwasi et al. (2017) [39]	United Kingdom	In-depth interviews (Narrative approach)	Ghanaians men and women aged 25–68 years (*N* = 31)	Recruited via gatekeeper, personal contacts, and snowballing	Dietary acculturation
Tiedje et al. (2014) [33]	United States	Focus groups (Community-based participatory research)	Somali, Mexican, Cambodian, and Sudanese immigrants, males and female, low SES, aged 11–65 years (*N* = 54)	Recruited via community partners using a purposive sampling method.	Dietary practices and healthy eating
Venkatesh and Weatherspoon (2018) [34]	United States	Focus groups (Qualitative study)	Indian immigrants, men and women, middle to high SES, aged 18 and over (*N* = 30)	Recruited via Indian clubs, temples, Indian stores, restaurants, and physicians’ offices using flyers	Dietary acculturation
Wilson and Renzaho (2015) [46]	Australia	Semi-structured interviews and focus groups (Exploratory qualitative)	Ethiopian, Somali and Sudanese refugees’ parents (*N* = 15 adolescents and 25 parents)	Recruited via gatekeepers such as ethnic organisations, clubs and church using purposive sampling method	Food beliefs

**Table 4:** Results of meta-synthesis of qualitative research findings

**Findings**	**Categories**	**Synthesised findings**
Familiarity	Socio-cultural factors	Social, cultural and religious factors
Cultural and racial well-being		
Positive behaviours		
Cultural identity		
Culture		
Loss of culture		
Reinforcing factor influencing being a South Indian		
Native” social and cultural understandings and influences of food-related behaviour from India		
Identity		
Community values and cultural identity		
Black ways of eating		
Social network		
Religious beliefs	Religious beliefs	
Family, community, and religious ties to traditional African foods		
Food, caring and happiness	Food for hospitality	
Social pressure		
Tradition of hospitality		
Hospitality and migrant context		
Enjoyable experiences	Psychological Factor	
Filling		
Hunger and feeling full		
Convenience	Convenience	Availability and accessibility
Shortcuts		
Busy lives		
Busy and complex lifestyles		
Convenience of Australian food		
Time	Time scarcity	
Cost	Cost Food preparation	
Cost and availability		
Socio-economic status		
Affordability		
Finances		
Poor access to home country foods		
Easy access to Canadian foods and prestigious foods		
Food insecurity and reliance on food banks		
Accessibility and availability		
Enabling factors in food choices and meal consumption patterns		
Availability		
Predisposing factors influencing being a South Indian		
Quality/freshness		
Restaurants		
Americanised eating habits		
Easy access to junk food		
Availability of traditional foods		
Access to traditional foods		
Varied eating patterns according to the availability and resources		
Food preparation		
Time for cooking traditional foods		
Family and community well-being	Family and community influence	Family and community influence
Extended family		
Children role		
Neighbourhood		
Living alone		
Living in households with a large group of people		
Nurturers; family, friends, and community		
Role of family		
Preservation of original food culture		
Attitudes and openness		
Preference for the traditional African Caribbean		
Taste and cravings		
Canadian versus Indian foods		
Traditional African cuisine is healthy and American foods as non- healthy Perception of the host country/traditional		
Differences between host foods and original food		
Perception of host country’s food	Lack of portion control	
Born and lived a long time in India before migration		
Cultural foods and traditions		
Born and lived in the United States		
Predisposing factors in food choices and meal consumption patterns	Dietary acculturation	Dietary acculturation
Changes in staple foods		
Migration and lifestyle changes		
Predisposing factors for adjusting to a Life in State College		
Sweets and deep-fried snacks		
Dietary practices following migration		
Reinforcing factor to adjusting to life in a State College		
Migration context		
Changes in meal pattern		
Dietary acculturation		
Loss of family commensality and identity, and purpose		
Change of preference over time		
Contrast between elders’ reliance on traditional Indian foods and young people’s desire for ‘western’ food		Dietary variations
Generational conflict	Generational variations and Dietary behaviours	
Bread spread		
Attitudes of family members		
Children’s preferences		
Acculturation: differences between generations in food preferences		
Second generation		
Dietary Fragmentation		
Nutritional quality	Health and healthy eating	Health and healthy eating
Healthy eating discourses		
Physical well-being		
Physical health		
Health		
Complementary foods (fish)		
Egg		
Aspects		
Intersection of “native” beliefs and American society and structures		
Reconciling conflicting interpretations about health risk		
Perception of healthy eating		
Healthy eating-meanings		
Healthy eating-motivations		
Family eating-habits		
Health and nutrition		
Food beliefs and perceived health consequences parents’ views		
Vegetables and legumes		
Complementary foods (Meat)		
Type of fats chosen		
Distrust in health professionals, nutrition and health promotion messages	Nutrition knowledge	Perception of nutritional information
Mistrust of providers		
Dominant biomedical dietary health meanings		
Recommendation of culturally inappropriate dietary advice	Culturally sensitive nutritional information	
Perception		
Culturally relevant and specific to African Americans		
Nutrition information		
People could eat healthfully if they knew what to buy		
Cooking skills		
Possible intervention		
Factors affecting dietary choices		
Exposure to host culture through the media and books		
Family counselling		
Community education		
Food purchasing		

### Perceptions of dietary intake among minoritised ethnic groups in high-income countries

#### Social and cultural factors

Beyond nutrients and health, social and cultural factors were shown to have an influence on Black, Asian and other minority ethnic groups’ food choices. The findings showed that individuals of Black, Asian or other minority ethnic background attach great importance to traditional foods because they view them as markers of their ethnic identity and membership within their specific ethnic and social network, as shown in Table 4. One participant in a study targeting the Ghanaian population in the UK [39] noted:

*“I am a traditional Ashanti man; [participant asks his daughter who was in the room]—what is my favourite food? [She answers fufu]—yes! Fufu, what the Ashanti man calls fufuo. You know what, until the day I die, that will be my favourite food”* (Ghanaian population, UK).

Food and food practices are commonly used to strengthen relationships among families, friends and communities. Traditional food consumption invokes good memories and connects a person to their cultural heritage. Traditional food is viewed as an important aspect of culture that must be preserved; therefore, it is paramount to transfer them from one generation to another. The use of food for hospitality and sharing food during festivals are also part of these food practices. For instance, Pakistani migrant women in Norway reported that a way of honouring guests is to serve them lots of food:

*“When you serve a lot of food, you honour your guests, and at the same time, you save your own reputation —You think badly of those that do not serve enough”* (Pakistani population, Norway).

Furthermore, hospitality has a religious undertone, especially among Muslims. Food is used as an offering to God, which illustrates people’s appreciation for God’s provision of abundant food and nourishment. Fasting is another common food practice among Black, Asian and other minority ethnic groups, which is used to offer reverence to God with the belief that it purifies and strengthens the individual. Other common cultural and religious beliefs among Black, Asian and other minority ethnic groups include vegetarianism or avoiding prohibited meats, such as beef or pork. However, individuals do not always strictly adhere to these beliefs due to the limited options available in the host countries. Psychological factors, such as enjoyment and fullness, were also given as the reasons for their food choices. Social and cultural meanings are crucial factors shaping Black, Asian and other minority ethnic groups’ food choices and food habits.

#### Availability and accessibility

Individuals of Black, Asian and other minority ethnic backgrounds are challenged by a lack of availability and accessibility of their traditional foods, which might impact their food intake and practices. They face challenges such as a lack of time to purchase and prepare their traditional foods, occasioned by changes in their daily routines following their migration from low-middle-income countries to high-income countries. These may have made them settle for convenience food, such as takeaway food, due to a lack of time for food preparation. Most individuals of Black, Asian and other minority ethnic groups indicated that more time is required to prepare traditional food than western food; therefore, it was more feasible for them to eat fast food (Table 4). This is illustrated by members of this population from Uganda and Zimbabwe living in Canada [35] and the UK [29], respectively:

*“In Uganda, [. . .] most people have maids in their home, so they don’t cook, they just make a demand. They just say, today, go buy this and this, they are not the ones going to do it. Here in Canada, you have your child; you have your house, you have your community; you have a lot in one person”* (Ugandan population, Canada).

* “Back home, for example, most families at home will have a cooked meal at lunch as well, the reason being there’s always a maid who is instructed to make the meal, but here because we have got to do it all ourselves and there’s just no time in the day to do it people end up skipping meals”* (Zimbabwean population, UK).

Another structural constraint that Black, Asian and other minority ethnic groups encountered was the cost of food; they believe that traditional and healthy foods are more expensive than unhealthy foods. Therefore, they were forced to buy unhealthy foods that were more affordable. For example, healthy foods like fruits and vegetables are perceived as more expensive and less available in predominantly ethnically diverse neighbourhoods. However, some Black, Asian and other minority ethnic communities acknowledged a recent increase in the availability of traditional foods. Another problem that ethnicity diverse groups face is the scarcity of fresh and good-quality food in their neighbourhoods, as those who rely on food banks indicated that they had no choice but to settle for low-quality foods that the food banks provide. The abundance in the availability of cheap junk food was a concern for Black, Asian and other minority ethnic groups and may be linked to the promotion of unhealthy eating among diverse ethnic groups. This usually occurs during the transition periods when the immigrants try to integrate into the new culture in their host countries.

### Family and community influence

Black, Asian and other minority ethnic communities prioritise family and community well-being over health when considering what food to eat. Family and community members can negatively or positively influence the food choices and adoption of healthy eating among diverse ethnic groups. Family members, especially children, were reported to play a pivotal role in the family’s food choices. Although women are usually in charge of food provision, they are not solely responsible for decision-making around food intake. Women wish to provide their family members with their preferred foods but do not have time to prepare different dishes. Therefore, they end up eating the same food as their family members. Healthy food, such as home-cooked food consisting of vegetables and legumes, is substituted for junk food and sweets to make their children happy. This was reported among Pakistani populations in Norway:

*“Vegetable curries are the first dishes to disappear from our food culture due to the fact that the children do not like it. [ ...] and next to go are legumes”* (Pakistani population, Norway).

Food has a pivotal role in social and religious gatherings such as festivals. Commensality practice, where a group of people eat together, is a popular practice that promotes solidarity and well-being in a community. Despite the positive association of commensality practice with family and community well-being, this practice was associated with over-eating and other related health problems. A large portion of traditional food, which consists of starchy, oily and salty meats, is served during festivals and community social gatherings. Moreover, it is socially inappropriate to refuse these good gestures. These practices promote unhealthy eating among ethnically diverse groups and predispose them to obesity and its related diseases. Family members can positively serve as positive change agents by encouraging their family to maintain aspects of their traditional food intake, such as vegetarian food.

Additionally, family members and religious groups, such as churches, have been shown to support individuals from Black, Asian and other minority ethnic groups in adopting healthy eating. Meanwhile, those living alone found it hard to maintain their traditional food habits. They found it easier to get fast food rather than prepare traditional food.

### Food preferences

Findings showed that across the included papers, individuals from Black, Asian and other minority ethnic backgrounds prefer their traditional food and will do anything to retain their food and food behaviours. They prefer their traditional food over Western food because their traditional food distinguishes them from other ethnic groups; their manner of eating is also linked to their history, especially among indigenous groups, such as African Americans and the African Nova Scotians in Canada [42].

*“We tend to like our fried foods, like fried chicken and potatoes and corn. And I find that we eat heavier, heavier meals. Like for instance, when I was growing up, I had White friends that I went to school with that I may go to their houses and have meals, and they may have like a chicken with the skin off it and potatoes, like not peeled, just whole potatoes. And maybe like some lemon sauce on it, really not much flavour. I think it is a lighter meal. ...I think we eat a lot, like our plates are pretty much stacked up ... Down home, you get a lot to eat”* (African Nova Scotian, Canada).

Eating traditional food is a way of connecting with their ancestors and remembering their forefathers during slavery. Although individuals from Black, Asian and other minority ethnic groups may prefer to continue eating their traditional food, they have usually had to adopt some aspects of Western food. When traditional food is unavailable, Black, Asian and other minority ethnic communities will try to find food in their host country that is similar to their original food as a substitute, which will enable them to preserve their original food culture.

### Home country food versus host country food

Black, Asian and other minority ethnic communities can view the host country’s food as not filling, whereas traditional food provides them with the strength to perform their daily jobs. This strength, they believe, translates into health:

*“Roti has a lot of strength in it. Dahl and subjee. They have a lot of strengths. … It is very good for the health. … Whatever I eat, I am able to digest, I am okay. It doesn’t bother me. So why should I have any concerns?”* (Punjabi Elder, Canada) [43].

There are conflicting opinions on the health benefits of their home country’s food versus the host country’s food; however, some Black, Asian and other minority ethnic communities believe that traditional food is healthier than the host country’s food because it is homemade and often served with vegetables. They believe that the host country’s food is less healthy because it is full of sugar and usually processed or genetically modified food. Many Black, Asian and other minority ethnic communities would classify takeaway food as host food, especially if the host country were their first encounter with takeaway food. In contrast, some individuals from Black, Asian and other minority ethnic communities, especially young adults, described traditional food as unhealthy and found the host country’s food healthier. They feel that traditional food is oily with no portion control, contributing to the higher risk of obesity and other non-communicable diseases among Black, Asian and other minority ethnic groups.

### Dietary acculturation

Dietary acculturation is the process by which immigrants adopt the dietary practices of the host country. The process of dietary acculturation includes adopting the host country’s food, flexibility and changing practices. The most-reported process of dietary acculturation is the flexibility process, in which Black, Asian and other minority ethnic groups combine both the home countries and the host country’s food. It was also observed that not only the types of food affected but also the food context and behaviours were eroded by dietary acculturation. For example, some ingredients for traditional food could be replaced with the host country’s ingredients, while the consumption of hot food was limited to late evenings. It was difficult to fix strict mealtimes; eating three times a day faded away, and the family mealtimes were replaced with solitary eating at different times among the family members. These changes were blamed on the time constraints and the changing role of women, who were housewives in their original countries but had to take up formal jobs in their host countries. This is illustrated in a study among the Somalian ethnic population in Australia [34]:

*‘’the lunch is not the way it used to be in Somalia because people [are] busy and kids have school and the lunchtime usually back home, we don’t have that culture [in Australia] anymore, you know. Lunchtime, people come together and eat together, sometimes it is happening here, but it is not the culture we used to have, its different now”* (Somalian population, Australia).

It is worth noting that Black, Asian and other minoritiy ethnic communities strive to retain their staple foods and food culture, especially the sense of commensality. Therefore, there is a cultural food pattern with a heavy reliance on dishes from their original food culture, and some meals, such as dinner on weekends or at religious celebrations, are more culturally loaded.

### Health and healthy eating

Most Black, Asian and other minority ethnic individuals understand the connection between healthy eating, good health and longevity. These individuals described healthy eating as eating a balanced diet, homemade food, fruits and vegetables, sandwiches, fewer carbohydrates, and reducing sugar intake and portion sizes. They also viewed healthy eating habits as avoiding junk food or snacks and cooking healthier by using less oil and salt. This perception is reported among the Pakistani/Bangladeshi population in the UK [29] :

*“I don’t like fried things - not too much. Try to avoid fried things. Don’t like too much oil”* (Pakistani/Bangladeshi population, UK).

Furthermore, the importance of eating fresh and unprocessed food was understood as part of healthy eating. Many admitted that they have a higher risk for many non-communicable diseases due to unhealthy eating. Some Black, Asian and other minority ethnic individuals said that they are trying to substitute unhealthy food for healthy food; for example, instead of red meat, they eat poultry meat or fish, and instead of butter, they use olive oil. They added that more exposure to bioscience nutrition in the host country encourages them to adopt healthy eating. The importance of physical activity was stressed, as some people believe that even if you eat high-calorie food, you can burn it off by working hard.

Some individuals from ethnically diverse communities believe that several traditional foods are medicinal; therefore, they categorised these foods as healthy. Unhealthy eating was also associated with obesity, diabetes, hypertension, and other nutritional-related diseases. Some individuals were prepared to adopt healthy eating for a positive self-image and desire for a slim body, while others viewed a thin body as part of Western culture, which may not be culturally appropriate for women from some communities. Certain Black, Asian and other minority ethnic communities preferred ‘thick’ bodies, as they are believed to have the ability to withstand hard times. This is seen in a study among the African populations in Canada [42]:

*“Whether other people want to realise it or not, certain things aren’t meant for Black folk. Certain things aren’t meant for Caucasians, right, it’s they’re not, and that has to do with the structure of their body ... I see numbers saying that African Americans are affected by this, and that, I believe it’s because we’re compromising who we are and what our culture is. And what we’ve done is we’re losing a part of ourselves by taking on someone else’s culture... We forget how we are supposed to eat, and we sort of start Westernising it, as some people put it, and that’s when we start having health problems. We start getting away from who we are and what it is that we would normally eat. I mean, some people are much healthier and bigger than they are when they’re small”* (African Nova Scotian population, Canada).

### Perception of nutritional information

There were conflicting results on the knowledge of nutrition among diverse ethnic groups. Some individuals from Black, Asian and other minority ethnic backgrounds were aware of the nutrition information, whereas some lacked knowledge. The factors that determined the degree of nutrition knowledge included the level of literacy, use of the host country’s language, the host environment, and the degree of exposure to nutrition resources. Some young adults, especially those with higher qualifications and who had been residents for a long time, were conversant with bioscience nutrition information. Older adults articulated traditional nutrition knowledge, classifying food as hot or cold. Some individuals, especially mothers, confessed that they lacked cooking skills for the host food and maintained their usual way of cooking. Alternatively, they looked for shortcuts by preparing unhealthy food. The media, internet, and word of mouth from acquaintances were their main sources for nutrition information, although they claimed this information could be conflicting and may negatively impact their dietary intake. They agreed that doctors and other health professionals were credible sources of nutritional information, but this was rarely or very briefly mentioned. For example, Punjabi population in Canada [43] noted:

“*Just that we should get information as to what is good and healthy to eat, especially in Indian food, as here we come to know a lot about the English food, as to what is good and nutritious but not for the Indian food. Like how good is dahl for us and how much should subjee be cooked. No one tells us these things*” (Punjabi population, Canada).

People from Black, Asian and other minority ethnic communities often lack skills on how to distinguish between credible and non-credible nutrition information, which may lead to misinformation and the appropriation of fake nutrition messages. These groups suggested recommendations included family and community nutrition counselling, training for the nutrition advisers on the traditional food and food culture among diverse ethnic groups, employing more individuals of Black, Asian and other minority ethnic backgrounds as nutrition advisers and incorporating traditional food in the nutrition information resources. Furthermore, they requested specific interventions, such as modifying traditional food to make them healthy while retaining the original taste, understanding the correct portion sizes of food, eating healthily on a low budget, and how to understand and implement food labels. In addition, some women would like to attend practical cooking programmes.

## Discussion

This qualitative review presented a wide range of findings on how Black, Asian and other minority ethnic groups living in HICs perceived their dietary intake. The included studies highlighted a diverse range of factors that influenced Black, Asian and other minority ethnic groups’ food choices and their uptake of healthy eating. Although the studies were conducted in different countries, the results showed consistency in findings that provided themes for a meta-synthesis. This review extracted 133 findings that were grouped into 18 categories and then used to generate eight synthesised findings.

Overall, this review found that Black, Asian and other minority ethnic communities are aware of the effects of unhealthy eating on their health, and some of them have nutritional knowledge, but social and cultural factors, including structural factors, were deterrents to their healthy eating behaviours. An important finding from this review is that some participants believed that nutritional information based on bio-medical science was intended for only White population groups and that it was antagonistic to their cultural and community well-being.

Social and cultural factors such as ethnic identity, cultural preservation, fond memories of their country of origin, and social networking were identified as important factors influencing the eating practices of diverse ethnic groups, a finding similar to previous reviews on factors influencing the food choices of ethnic minorities living in high-income countries [4, 20, 47, 48]. This systematic review found that people of Black, Asian and other minority ethnic backgrounds living in HICs valued their traditional foods because they see them as a means of ethnic identity and social network, providing them with pleasant childhood memories. For example, some participants used traditional foods to distinguish themselves from white ethnic groups. Results from several studies in this systematic review [32, 39, 41, 42, 46] indicated that traditional foods are used as a means of group cohesion and a channel to resist racism.

Food availability and accessibility were consistently reported as other important factors influencing dietary practices found in the included papers in this review. Factors such as time, cost, poor availability of traditional foods and easy access to unhealthy foods have influenced Black, Asian and other minority ethnic population food practices. Time constraints resulting from combining a busy working schedule and family commitments were identified as barriers to maintaining traditional food practices and healthy eating. Food costs also impacted some participants’ healthy eating [33, 37, 41, 46]. Mellin-Olsen and Wandel [30] found that Pakistani immigrant women in Norway reported increased meat consumption due to increased affordability. Very few studies assessed participants’ social-economic status by using the combination of educational level, occupation, and household income. Other studies that reported the influence of cost on food practices failed to report the participants’ socioeconomic level [38, 49]. Poor availability of traditional and healthy foods and the preponderance of junk foods in the neighbourhoods have been reported to have impacted the food practices amongst ethnically diverse communities [27, 30, 33–35, 37, 39].

This review showed that children and second-generation immigrants are more likely to adopt host countries’ dietary practices faster compared to their parents [30, 32, 33, 39, 44, 45]. Previous reviews have also reported a similar pattern of dietary acculturation among immigrants in the HICs. Evidence has also shown that immigrants’ dietary practices change as they adapt to the dietary culture of the host countries [4, 11, 50, 51]. The majority of dietary changes were reported to be unhealthy food practices. However, the rate of dietary acculturation is influenced by age and immigration generation. Although in this review, many articles reported that participants expressed their preferences for traditional foods and they would like to continue traditional dietary practices; they were hindered by time constraints, lack of ethnic food stores, and the high cost of traditional food [27, 32, 35, 39, 45].

The family and community were also found to impact food practices among ethnically diverse groups; these could be facilitators or barriers to maintaining traditional food and healthy eating practices among Black, Asian and other minority ethnic groups. Family and community members, particularly children, influence participants’ food practices as some participants reported eating unhealthy foods to accommodate their children’s new food habits [30, 35, 38]. However, the quest to preserve the culture and secure family unity made some individuals from Black, Asian and other minority ethnic backgrounds encourage their children to eat traditional food rather than consuming host country foods. Furthermore, unhealthy food practices are common during cultural and religious festivals, and these practices are valued for community well-being [27, 32]. Sometimes, family and community can positively impact their food practices; for instance, participants living with family members could maintain their traditional food practices, while community and religious groups could be assets for promoting healthy eating [33, 42, 46]. This finding is similar to Ngongalah and colleagues [48], whose review on migrant women in HICs classified community and social networks as facilitators for maintaining traditional food practices.

Factors identified as facilitators of healthy eating in this review include social and cultural factors, nutritional knowledge, self-image, family and community support, growing old and ill health, while barriers to healthy eating include the higher cost of healthy foods, poor availability of healthy foods, an abundance of unhealthy foods, time constraints, familiarity, and food preference. These factors are consistent with the findings of previous reviews on healthy eating among the majority ethnic population in the HICs that highlighted sociocultural environment, structural environment, and individual characteristics like nutritional knowledge and skills, beliefs, and health as influencing factors to healthy eating [52–54].

Factors that are unique to this review are self-image and growing old; these factors have not been reported in previous reviews. Factors such as social marketing and food policies captured in the previous reviews among the majority ethnic groups in the HICs [52–54] were not eminent in this review. Most researchers investigating ethnically diverse dietary practices, particularly in the studies included in this review, focused more on sociocultural, availability and accessibility factors. This might be a bias in studies conducted among ethnically diverse groups.

Findings in this review suggest a conflicting report on levels of nutritional knowledge among participants. Some studies reported that individuals from Black, Asian and other minority ethnic backgrounds lack or have insufficient nutritional knowledge, while others lack the skills to cook healthy foods [30, 33, 35, 46]. Many participants perceived nutritional guidelines as inappropriate for them and an imposition of dietary rules that do not conform to their cultural way of eating. Individuals from Black, Asian and other minority ethnic backgrounds confessed that they lack healthy cooking skills, use a lot of oil for traditional foods and commonly consume large portions [28, 33, 49]. Also, the use of unconventional sources for nutritional information, such as social media, the internet and word of mouth, was commonly reported by groups. Therefore, Black, Asian and other minority ethnic groups may benefit from information on serving smaller portions of foods, healthy cooking programmes, understanding food labels, and identifying good sources of nutrition information. Participants also advocate for more Black, Asian and other minority ethnic community members to be employed as nutrition advisers.

Most participants claimed they could not adopt healthy diets because they could not afford to purchase them [27, 30, 41, 42, 46]. Evidence has shown that healthy foods are more expensive than unhealthy foods [55–58]. High levels of social inequality exist among ethnically diverse groups, which is seen in a country like the UK, where a high proportion of Black, Asian and other minority ethnic groups live in poverty [59, 60]. Due to financial constraints, some individuals may be unable to afford healthy diets. Therefore, there is a need to tackle social and health inequalities among minority groups; government policies to reduce the cost of healthy foods and availability of unhealthy foods will help the ethnically diverse communities living in the HICs to adopt healthy eating and reduce the risk of obesity and other nutrition-related diseases.

This review also indicates that family and community negatively and positively influence dietary intakes [27, 35, 38, 42, 46]. Family and community organisations such as religious and cultural institutions can be incorporated into healthy eating interventions targeted at the Black, Asian and other minority ethnic groups to achieve success and ensure the sustainability of healthy eating among the minority ethnic group.

### Strengths and limitations of the review

The strength of this review is the assessment of the Black, Asian and other minority ethnic groups’ levels of nutrition knowledge and their perceptions of nutrition guidelines. Understanding the perceptions of nutrition guidelines among ethnically diverse groups can help inform recommendations to formulate culturally sensitive healthy eating resources and promote healthy eating and healthy weight among these population groups. In the same vein, the methodological strengths of this review include the rigour applied to the systematic search of several databases. This ensured that the originality of the findings was protected and made possible the generation of statements that may inform policy and practice in promoting healthy eating and preventing obesity among ethnically diverse groups. Also, JBI standardised quality appraisal was used to ensure high-quality studies were included in this review. The limitation of this review is that only studies published in the English language were included; therefore, other relevant studies published in other languages could have been missed. Although an extensive literature search was conducted in the electronic databases with specific keywords, we might have missed some articles. In the same vein, the study did not include some parameters around socio-demographic factors and medical and migration history of the participants reported in the included studies because this information was not provided in the eligible articles.

### Implications and recommendations for future research

There is a need for future methodologically strong studies that will provide objective data on dietary intakes across different ethnic groups. Additional research with empirical data on nutrition knowledge and usage of nutrition guidelines is needed to supplement the findings presented in this review. This review identified gaps in studies on dietary practices among ethnically diverse groups in HICs since a small fraction of the studies reported conflicting reports on nutritional knowledge among Black, Asian and other minority ethnic groups, and the majority of the articles did not report on the usage of healthy eating resources. Future studies should use a standardised instrument to measure food intake among ethnically diverse groups and assess the nutritional components of traditional foods. Having in-depth knowledge of the traditional foods’ components may inform the development of culturally sensitive dietary resources.

## Conclusions

This review presents evidence on the dietary intake perceptions of Black, Asian and other minority ethnic groups living in high-income countries; the findings indicate that social and cultural factors and availability and accessibility of foods influence dietary practices among ethnically diverse groups. There were conflicting reports on the influence of SES levels and food practices. Some studies found low SES to be associated with unhealthy dietary intake; in contrast, other studies found no association between SES and dietary practices. In addition, reports on levels of people’s nutritional knowledge varied across the studies. While some studies found that Black, Asian and other minority ethnic groups had good nutrition knowledge, others reported contrary findings. Identified gaps in the research include a need to conduct a study in the UK that will explore the underlying mechanisms that shape dietary practices among ethnically diverse groups and which can be used to develop culturally sensitive nutrition guidelines that are more likely to be accepted and implemented. Future research should address factors influencing the Black, Asian and other minority ethnic groups’ healthy eating.

## Data Availability

The datasets used and/or analysed during the current study are available from the corresponding author upon reasonable request.
